# Analysis of the Factors Affecting the Interval between Blood Donations Using Log-Normal Hazard Model with Gamma Correlated Frailty

**Published:** 2014-06-12

**Authors:** Najmeh Tavakol, Soleiman Kheiri, Morteza Sedehi

**Affiliations:** ^a^ Student's Research Committee, Shahrekord University of Medical Sciences, Shahrekord, Iran; ^b^ Social Health Determinants Research Center, Shahrekord University of Medical Sciences, Shahrekord, Iran; ^c^ Department of Epidemiology and Biostatistics, School of Public Health, Shahrekord University of Medical Sciences, Shahrekord, Iran

**Keywords:** Blood Donation, Correlated Frailty, Recurrent Event, Log-Normal Hazard Model, Bayesian Method, Markov Chain Monte Carlo

## Abstract

**Background:** Time to donating blood plays a major role in a regular donor to becoming continues one. The aim of this study was to determine the effective factors on the interval between the blood donations.

**Methods:** In a longitudinal study in 2008, 864 samples of first-time donors in Shahrekord Blood Transfusion Center, capital city of Chaharmahal and Bakhtiari Province, Iran were selected by a systematic sampling and were followed up for five years. Among these samples, a subset of 424 donors who had at least two successful blood donations were chosen for this study and the time intervals between their donations were measured as response variable. Sex, body weight, age, marital status, education, stay and job were recorded as independent variables. Data analysis was performed based on log-normal hazard model with gamma correlated frailty. In this model, the frailties are sum of two independent components assumed a gamma distribution. The analysis was done via Bayesian approach using Markov Chain Monte Carlo algorithm by OpenBUGS. Convergence was checked via Gelman-Rubin criteria using BOA program in R.

**Results:** Age, job and education were significant on chance to donate blood (*P*<0.05). The chances of blood donation for the higher-aged donors, clericals, workers, free job, students and educated donors were higher and in return, time intervals between their blood donations were shorter.

**Conclusions:** Due to the significance effect of some variables in the log-normal correlated frailty model, it is necessary to plan educational and cultural program to encourage the people with longer inter-donation intervals to donate more frequently.

## Introduction

Blood and blood products play a big part in saving patients’ lives. Regarding the increase in the consumption of blood because of various reasons, it is necessary that donors increase as well^1^. Providing sufficient and healthful blood is up to blood transfusion centers. Unless enough healthy blood is provided, the society’s health will be at stake. Therefore, those in charge of health care in society are looking for ways of supplying blood and preventing lack of it in blood banks. For this end, detecting and attracting constant donors is of great importance^2^. Constant blood donors, according to the standards of the Blood Transfusion Organization, are those who donate blood at least twice a year. Therefore, the blood transfusion centers try to increase the number of constant donors in order to ample blood supply for the patients in need of it^3^.

When it is possible for a person to experience something several times, a recurrent event takes place. The data obtained from the repetition of these events are called recurrent events data. Blood donation is considered a recurrent event in survival analysis^4^. In recurrent events survival data, due to individual differences and the effect of previous events, there is a correlation between survival time intervals^5^. Owing to the correlation between the independent survival recurrent times, hazard regression models with independent data cannot be applied and it is necessary that a model capable of covering this correlation is used^6-8^. One way of handling this problem is to use a frailty models. In this model, a common random effect called frailty effect is given to the members of each group later multiplied in the hazard function of each group member ^8^.

Since the log-normal hazard model with correlated frailty is very complex, Bayesian approach was used to estimate parameters of this model. In this approach, the prior knowledge about the parameters and likelihood is used to produce the posterior distribution, which represents total information about the parameters after the data have been observed^9^. Due to the complexities of frailty models, it is not possible to calculate the posterior distribution of the parameters in an analytic way^10^. Therefore, in order to do a Bayesian analysis, it is necessary to estimate the posterior distribution of parameters using Markov Chain Monte Carlo (MCMC) method. Successive sampling of full conditional distributions of parameters produces a Markov Chain; so that after convergence these samples can be assumed as dependent samples from marginal posterior distributions of parameters and based on them inference about parameters of interest can be done. In recent years, by using MCMC methods, complex and extensive models for any sample size have been conducted and precise estimations have been yielded^11^.

Log-normal hazard model is one of important applicable model in survival analysis which belongs to the generalized gamma parametric survival model^12^. In the log-normal model, the hazard rate increase from zero rapidly to a peak and then decrease gradually, so that it is a unimodal with comparatively long tail in the right^13, 14^. In a primary study on some parametric models without frailty, the log-normal model showed a better fit on our data compare to exponential, Weibull, log-logistic and generalized gamma models. Getting the log-normal model to fitting the interval between blood donations is reasonable because immediately after a successful donation, the chance to another donation is zero, then the donation chance increase with time, and after passing a long time from previous donation, the chance decline.

The aim of this study was to determine the effective factors on the interval between the blood donations in a sample consisting of first-time blood donors based on log-normal hazard model with correlated frailty.

## Methods

A series of unknown factors can influence the time intervals between donations including donor's related and specific donation related. Therefore, a correlation between each person’s survival times is expected, which is the time interval between two donations for that person. Accordingly, a random variable called correlated frailty is considered for each person in order to recognize the correlation of the time interval between two donations of that person then the hazard function is multiplied^16^. The correlated frailty (Yij) consists of shared frailty (Wi) plus individual frailty (Zij), which are independent from each other; moreover, by considering their values, it can be said to what extent the survival time of each sample has been influenced by either common unknown factors or specific ones^16^. In this study, it was supposed that frailties were of gamma distributions with θ scale; hence, given the independent nature of frailty terms from each other, correlated frailties will also have gamma distributions. As a result, shared frailty is of a gamma distribution with φ,θ parameters and individual frailty is of a gamma distribution with θ-φ,θ parameters provided that 0<φ<θ. Then, the correlated frailty (Yij) has a gamma distribution with θ,θ parameters with mean one and variance of θ-1. Since the survival times for each person are correlated, the correlated frailty model was used. Therefore, regarding the frailty in the model, the survival times for each person were supposed to be independent (from each other)^16^. A full presentation of correlated frailty model for baseline log-normal hazard model is given in the appendix.

In order to modeling the baseline hazard function, log-normal hazard model was used. For analyzing return to donation, data three models were used as log-normal hazard model without frailty, log-normal hazard model with gamma-shared frailty, and log-normal hazard model with gamma correlated frailty. Furthermore, the estimation of parameters was done using Bayesian analysis and Markov Chain Monte Carlo (MCMC) method. Considering the complexity of posterior distribution calculations, MCMC method is used to estimate the parameters for any sample size^11^.

For prior distribution of the model parameters, first the log-normal hazard model applying non-informative prior of regression coefficient was carried out and later their estimate was used for determining informative prior distribution in the shared frailty and correlated frailty models. Therefore for the parameters of regression, the prior for the coefficient of age assume as normal distribution with mean 1 and variance 100, sex as normal distribution with mean 1 and variance 4, body weight as normal distribution with mean 0 and variance 100, education as normal distribution with mean 1 and variance 4, job as normal distribution with mean 1 and variance 4, marital status as normal distribution with mean 0.1 and variance 4, stay as normal distribution with mean 1 and variance 2 and a β0 (constant value) as normal distribution with mean 1 and variance 4 were used. The prior distribution for frailty parameter of (φ) as uniform distribution on (0,θ) and for frailty parameters of (θ) as gamma distribution (α=0.5,β=10) were used as well. The parameters were estimated using OpenBUGS^17^ software version 3.2.3. In order to make sure of the coverage of Monte Carlo simulations, Gelman-Rubin convergence criteria via BOA (Bayesian Output Analysis) program in R software version 3, 2, 0 was used^18^. Comparing between log-normal hazard model without frailty, log-normal hazard model with gamma shared frailty, and log-normal hazard model with gamma correlated frailty was done based on deviance information criterion (DIC)^19^.

## Results

**Table 1:** Some characteristics of donors in the study (n= 424)

**Continuous variables**	**Mean (SD)**
Age (yr)	36.50 ±10.20
Body weight (kg)	80.16 ±11.63
**Categorical variables**	**Number (%)**
Gender	
Male	404 (95.3)
Female	20 (4.7)
Marital Status	
Single	118 (27.8)
Married	306 (72.2)
Residential location	
Urban	344 (81.9)
Rural	80 (18.9)
Occupation	
Housewife	13 (3.1)
Clerical	101 (23.8)
Worker	59 (13.8)
Free Job	192 (45.3)
Student and Unemployed	59 (13.9)
Educational level	
Elementary school	69 (16.3)
High School	102 (24.1)
Diploma	145 (34.2)
Academic level	108 (25.5)

The time between recurrent donations was a response variable. The number of donations in each interval, the mean of time interval between donations and the censoring rate for each time interval is given in Table 2.

**Table 2:** Summary information of blood donation interval

**Blood donation interval**	**Number of** **donation**	**Censor rate** **of interval (%)**	**Survival time** **Mean ±SD (day)**
First	424	34.0	555 ±432
Second	280	38.2	438 ±365
Third	173	36.4	317 ±278
Fourth	110	32.7	242 ±199
Fifth	74	29.7	203 ±144
Sixth	52	23.1	162 ±127
Seventh	40	35.0	170 ±128
Eighth	26	26.9	157 ±990
Ninth	19	31.6	166 ±980
Tenth	13	69.2	172 ±117
Eleventh	4	75.0	176 ±145
Twelfth	1	1.0	314 ±000

The results of fitting the log-normal hazard model with gamma correlated frailties, including mean, median, standard deviation, and 95% credible intervals, based on 30,000 simulated values after considering 5000 samples as burn-in period, is shown in the Table 3. Since there was a very high autocorrelation in the successive values of the simulated observation, every 50-th sample was monitored. The estimate of Gelman-Rubin convergence criteria is shown in the last column of Table 3. These values are very close to one ensuring the convergence of all parameters of model. In our data, DIC for log-normal hazard model without frailty was 32814800, for the log-normal hazard model with gamma shared frailty was 32810000, and for the log-normal hazard model with gamma correlated frailty was 32210000, which showed a better fitting for the log-normal hazard model with gamma correlated frailty. Based on estimate of φ and θ parameters, the estimate of variance of frailty random effect is 0.41, the mean of shared frailty is 0.66, the mean of individual frailty is 0.34, and the coefficient correlation of shared and individual frailty is 0.66.

**Table 3:** Posterior summaries for the parameters of log-normal correlated frailty model

**Parameter **	**Mean **	**Median **	**SD**	** Chain Error **	**95% CI **	**Gelman-Rubin Criteria**
Age (yr)	0.012	0.012	0.004	0.0001	0.004, 0.020	1.000
Education Level High School vs. Elementary	0.097	0.099	0.120	0.005	-0.141, 0.333	1.002
Diploma vs. Elementary	0.257	0.259	0.120	0.005	0.025, 0.491	1.000
University vs. Elementary	0.321	0.320	0.133	0.004	0.064, 0.584	1.000
Job worker vs. housewife	1.075	1.065	0.234	0.012	0.647, 1.548	1.000
Clerical vs. housewife	1.040	1.024	0.240	0.013	0.612, 1.569	1.003
Free Job vs. housewife	1.189	1.178	0.229	0.012	0.775, 1.691	1.003
Student and Unemployed vs. housewife	0.640	0.627	0.237	0.012	0.201, 1.144	1.006
Marital status(Single)	0.071	0.071	0.069	0.002	-0.065, 0.207	1.000
Sex(Male)	-0.061	-0.060	0.232	0.011	-0.520, 0.390	1.001
Stay(Village)	0.136	0.135	0.083	0.002	-0.030, 0.303	1.001
Body Weight(kg)	-0.004	-0.004	0.003	0.001	-0.011, 0.002	1.003
Constant	4.260	4.272	0.299	0.015	3.666, 4.798	1.002
Parameter θ	2.419	2.286	0.649	0.054	1.638, 4.499	1.005
Parameter φ	1.614	1.428	0.782	0.069	0.744, 4.290	1.002
τ	0.946	0.946	0.068	0.004	0.811, 1.081	1.001

## Discussion

In the transfusion data framework, the correlated effect is sum of donor random effect (known as shared frailty) and specific donation random effect (known as individual frailty). The mean estimate of donor random effect in our data was almost 2 times of specific donation random effect. This results show that effect of donor's unknown factors are higher than specific donation's unknown factors. The correlation of interval times between donations of each donor means that each donor returns to donation in a roughly equal interval which show stability in his/her return to donation behavior.

The correlated frailty was used in many applications,^28^. Here we used it to explain unknown random effect in the recurrent event survival data. Log-normal hazard model for survival analysis is complex especially in present of censoring,^13^. Its complexity increased by applying the random effect in the model. This problem causes the difficulty in convergence of simulated samples and encountering a high autocorrelation between subsequent samples of simulated values. In running of MCMC algorithm, one sample was monitored from every 50 produced samples to reduce autocorrelation between subsequent samples and obtaining convergence. Although a large development in computational speed has been created, Bayesian analyzing of such model is very time-consuming presently.

In this study, it was presupposed that components of frailty were of gamma distribution. Research can also be done on log-normal hazard model with correlated frailty having Inverse Gaussian and Positive Stable distributions.

## Highlights

- The effects of donor's unknown factors are higher than specific donation's unknown factors.
- Interval times between donations of each donor are correlated.
- In our survival data, the correlated frailty showed better fitting than shared frailty

## References

[1] Grindon AJ, Newman B. Blood donation. In: Hillyer‏ CD, Silberstein LE, Ness PM, Anderson KN, Roback‏ JD. Blood banking and transfusion medicine: Basic principles and practice. Philadelphia: Churchill‏ Livingstone; 2003.

[2] Salehi H, Salehi M, Khish Ardestani M, Khorvash F, Mostafavi Zadeh K (2011). Comparing the‏ blood safety on the blood donors within the religious ceremonies and routine conditions. J Isfahan Med Sch.

[3] Mahdaviani F, Saremi S (2005). ‏ The study of the regular donors as members of recruitment‏ unit of Arak Blood Transfusion Center in the first‏ six months of the year 1382 (2004). IBTO-Research Center.

[4] Cook RJ, Lawless JF. The Statistical Analysis of Recurrent Events. New York: Springer; 2006.

[5] Kelly PJ, Lim LL (2000). Survival analysis for recurrent event data: An application to childhood infectious disease. Stat Med.

[6] Henderson R, Oman P (1999). Effect of frailty on marginal regression estimate in survival analysis. J Am Stat Assoc.

[7] Keiding N, Andersen PK, Klein PJ (1997). The role of frailty models and accelerated failure time models in describing heterogeneity due to omitted covariate. Stat Med.

[8] Clayton DG (1978). A model for association in bivariate life table and its application in epidemiological studies of chronic disease incidence. Biometrika.

[9] Bolstad WM. Introduction to Bayesian Statistics. New Jersey: John Wiley & Sons; 2013.

[10] Ibrahim JG, Chen MH, Sinha D. Bayesian Survival Analysis. New Jersey: John Wiley & Sons; 2005.

[11] Congdon P. Applied Bayesian modeling, 2nd ed. New Jersey: John Wiley & Sons; 2014.

[12] Newcombe PJ, Ali HR, Blows FM, Provenzano E, Pharoah PD, Caldas C, Richardson S. Weibull regression with Bayesian variable selection to identify prognostic tumour markers of breast cancer survival. Stat Methods Med Res. 2014: 0962280214548748. 10.1177/0962280214548748 PMC6055985 25193065

[13] Makkar P, Srivastava PK, Singh RS, Upadhyay SK (2014). Bayesian survival analysis of head and neck cancer data using log-normal model. Commun Stat Theory Methods.

[14] Royston P (2001). The log-normal distribution as a model for survival time in cancer, with an emphasis on prognostic factors. Stat Neerl.

[15] Kheiri S, Alibeigi Z (2015). An analysis of first-time blood donors returns behavior using regression models. Transfus Med.

[16] Hougaard P. Analysis of Multivariate Survival Data, New York: Springer; 2000.

[17] Spiegelhalter DJ, Thomas A, Best N (2009). The BUGS project: Evolution, critique and future direction. Stat Med.

[18] Gelman A. Inference and monitoring convergence. In: Gilks WR, Richardson S, Spiegelhalter DJ. Markov chain Monte Carlo in practice. London: Chapman and Hall; 1996, pp 75-88.

[19] Spiegelhalter DJ, Best NG, Carlin BP, Van Der Linde A (2002). Bayesian measures of model complexity and fit. Series B.

[20] Ownby HE1, Kong F, Watanabe K, Tu Y, Nass CC (1999). Analysis of donor return behavior Retrovirus Epidemiology Donor Study. Transfusion.

[21] Wu Y, Glynn SA, Schreiber GB, Wright DJ, Lo A, Murphy EL (2001). First-time blood donors: Demographic trends. Transfusion.

[22] Jahangiri Mehr F, Kheiri S, Sedehi M (2015). Bayesian analysis of the factors affecting the interval between blood donations using Cox's shared frailty model: A longitudinal study on a sample of blood donors in Iran. J Health Syst Res.

[23] James RC, Matthews DE (1996). Analysis of blood donor return behaviour using survival regression methods. Transfus Med.

[24] Massaeli Z, Jaberi MR, Hariri MM (2007). Effect of recruitment of first time blood donors on donor return behavior in Isfahan. Sci J Iran Blood Transfus Organ.

[25] Hasanzadeh A, Farahini Farahini, F F, Akbari N, Aghahosseini M, Pirzadeh A (2013). Survey of effective factors on continuous blood donation in Isfahan province based on the theory of planned behavior. Sci J Iran Blood Transfus Organ.

[26] Kasraian L, Tavassoli A (2012). Relationship between first-year blood donation, return rate for subsequent donation and demographic characteristics. Blood Transfus.

[27] Kheiri S, Meshkani MR, Faghihzadeh S (2005). A correlated frailty model for analyzing risk factors in bilateral corneal graft rejection for Keratoconus: A Bayesian approach. Stat Med.

[28] Hanagal DD, Pandey A, Ganguly A. Correlated gamma frailty models for bivariate survival data. Commun Stat Simul Comput 2016; In press.

[29] Kalbfleisch JD, Prentice RL. The Statistical Analysis of Failure Time Data. 2nd ed. Hoboken: John Wiley & Sons; 2011.

